# Mandible evolution in the Scarabaeinae (Coleoptera: Scarabaeidae) and adaptations to coprophagous habits

**DOI:** 10.1186/s12983-015-0123-z

**Published:** 2015-10-28

**Authors:** Ming Bai, Sha Li, Yuanyuan Lu, Haidong Yang, Yijie Tong, Xingke Yang

**Affiliations:** Key Laboratory of Zoological Systematics and Evolution, Institute of Zoology, Chinese Academy of Sciences, Box 92, Beichen West Road, Chaoyang District Beijing, 100101 People’s Republic Of China; University of Chinese Academy of Sciences, Yuquan Road, Shijingshan Beijing, 100039 P. R. China

**Keywords:** Coprophagy, Dung beetle, Mouthparts, 3D, Geometric morphometric

## Abstract

**Introduction:**

The astonishing spectrum of scarabaeine lifestyles makes them an attractive group for studies in entomology and evolutionary biology. As a result of adaptions to specific food substrates and textures, the mouthparts of dung beetles, particularly the mandible, have undergone considerable evolutionary changes and differ distinctly from the presumptive ancestral conditions of the Coleoptera and Polyphaga. The possible functions of dung beetle mouthparts and the evolution of dung feeding have been controversial for decades.

**Results:**

In this study, 187 scarabs representing all tribes of the Scarabaeinae and the major lineages within the Scarabaeoidea, along with three major feeding types within the Scarabaeoidea (omnivory, phytophagy and coprophagy), were studied. Based on geometric morphometric and three-dimensional (3D) reconstruction approaches, morphological differences in mandibles among the three feeding types were identified. The ancestral forms of the mandible within the Scarabaeinae were reconstructed and compared with those of modern species. The most recent common ancestor of the Scarabaeinae fed on soft materials, and the ancestor of the Scarabaeinae and the Aphodiinae was in an evolutionary transition between processing more solid and softer substrates.

**Conclusions:**

Coprophagy originated from omnivorous ancestors that were very likely saprophagous. Furthermore, phytophagy may also have originated from omnivory. In addition, our study addresses the integration and modularity of geometric morphometric data in a phylogenetic context.

**Electronic supplementary material:**

The online version of this article (doi:10.1186/s12983-015-0123-z) contains supplementary material, which is available to authorized users.

## Introduction

Decomposers use deceased organisms and non-living organic compounds as their food source. By breaking down dead material, they provide nutrients that are crucial to the environment and essential for the survival of other organisms. Many species of bacteria, fungi and protists, the primary decomposers, are unable to ingest discrete masses of matter, but instead absorb and metabolize resources on a molecular scale. Insect decomposers, such as dung beetles, burying beetles, fly maggots, and others generally consume larger quantities of organic matter [1]. Vertebrate dung is a special niche that is considered highly desirable and nutritious to potential dung colonizers. A typical dung community consists of dung feeders or predators, many of which are beetles. These groups include species of the scarabaeoid Scarabaeinae, Aphodiinae, Geotrupidae, and Hybosoridae, and the hydrophiloid Sphaeridiinae, which use dung as a food substrate, but also include predaceous beetles belonging to the Staphylinidae or Histeridae. The true dung beetles belong to a single taxonomic entity and clade, the scarabaeid subfamily Scarabaeinae, which consists of nearly 6000 described species grouped into 240 genera [2]. The very broad spectrum of scarabaeine lifestyles, including elaborate nesting behaviors and specialized food preferences, has long fascinated insect researchers [3, 4].

**Fig. 1:**
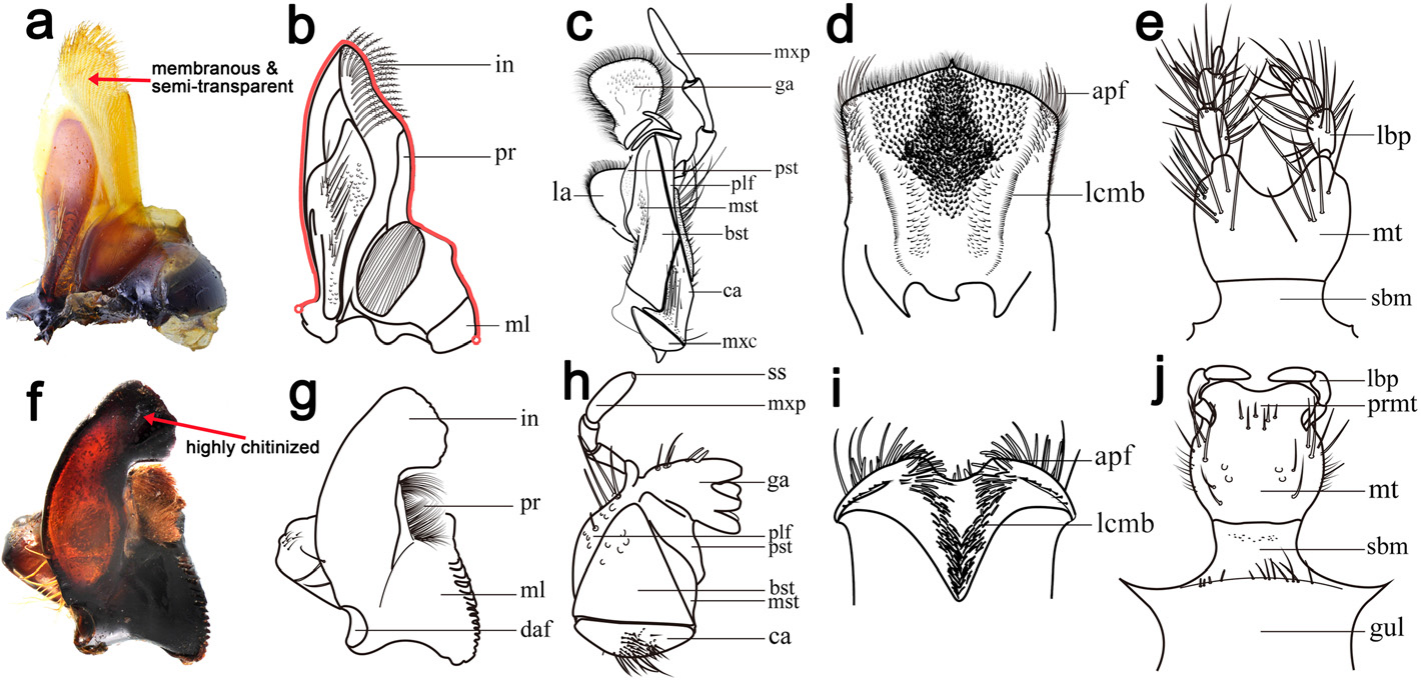
Mouthparts of coprophagous and phytophagous scarabs. Mandible (**a**, **e**); maxillary (**b**, **f**); epipharynx (**c**, **g**); labium (**d**, **h**). Coprophagous scarabs (**a**–**d**) represented by **a**: *Synapsis yunnanus*, **b**: *Heliocopris dominus*, **c**: *Onthophagus (Palaeonthophagus) gibbulus*, and **d**: *Paracopris punctulatus.* Phytophagous scarabs (**e**–**h**) represented by *Mimela passerinii mediana*. One curve (=50 landmarks) was selected from the mandible (**a**) for the geometric morphometric analysis

Adult and larval dung beetles are primarily coprophagous. However, their feeding habits are not limited to this substrate. Many species are known to specialize on carrion, the fruiting bodies of basidiomycote fungi, freshly dead millipedes [5, 6], rotting fruit and leaves, or the debris of attine leaf-cutter ants. There are even species that are predators of millipedes [7], reproductive ants, and termites. All of these food substrates are more or less soft and contain significant moisture. As a result of adaptions to food texture, the mouthparts of dung beetles have deviated considerably from the basic structure of the Coleoptera. In general, the major components of the Scarabaeinae mouthparts tend to be membranous and hairy (Fig. 1). The most variable element shows greatly weakened mandibular incisor lobes that are poorly adapted for the cutting of hard food. In addition, fine but robust ridged molar areas with a convex surface fitting tightly into the opposing concave surface are present in these mandibles [3, 4]. The morphology of the other mouthparts is not significantly different from the basic structure of the Coleoptera, except that the labrum-epipharynx is membranous and hairy, the maxillae is lacking a hook or tooth on the lacinia and there are strong paraglossae and very weak glossae in the labium. The membranous and hairy elements remove large fragments from the food and scoop liquid components into the oral cavity [8].

The possible functions of the dung beetle mouthparts have been a subject of controversy for decades (Fig. 1). It was presumed that the molar lobes crush large particles into much finer ones that are then imbibed [3]. However, trituration of dung particles was not confirmed in a serial experiment by Holter [9–13]. Moreover, Holter [9] assumed that the food was collected by the maxillary palps, that the large particles were brushed out by filtration setae on the mouthparts (primarily the maxillae and labium), and that the remaining paste was then squeezed by the molar lobes while the superfluous liquid was drawn away from the pharynx through filtration channels. The compacted material formed from compressed small particles was then ingested.

One proposed evolutionary scenario is that adult Scarabaeoidea shifted from harder and drier substrates (i.e., detritus) to a softer and moister diet (dung) [3]. Although there are some discrepancies in the detailed evidence presented [4], it is generally agreed that dung beetles likely evolved from a detritus-feeding ancestor, a hypothesis that is based on qualitative observations [14]. To date, quantitative analyses, including an approach using geometric morphometrics, have been lacking [15–17]. To test this scenario and to reconstruct the evolution of the character system, we selected the mandible, the most derived mouthpart component, from 187 species representing all major lineages of the Scarabaeoidea, with comprehensive sampling from the Scarabaeinae, for this study (Additional file 1: Table S1). Three feeding types (omnivory, phytophagy and coprophagy) were evaluated, and only the Scarabaeinae (true dung beetles) were treated as coprophagous (see Materials and methods for details). The mandibles associated with the three feeding types were initially compared based on two-dimensional (2D) and 3D morphological observations. The morphological variations of the mandible of studied species were then analyzed using geometric morphometrics. The ancestral form of the mandible for the studied species was reconstructed. Last, based on the obtained results, the evolution of the mandibles, which play a crucial role in feeding behavior, was inferred, and coprophagous adaptations were discussed. Our study also addresses the integration and modularity of geometric morphometric data in a phylogenetic context.

## Materials and methods

### Taxa examined

This study is based on exemplars of 187 species, most of which are housed at the Institute of Zoology, Chinese Academy of Sciences, and additional photographs of mandibles of species from the literature (Additional file 1: Table S1). The specimens were examined and dissected using a LEICA MZ 12.5 dissecting microscope.

The Scarabaeinae are described as true dung beetles because they exclusively use a dung-type soft food, such as dung, carrion, fungi, and other types. The mandibles of scarabaeines are typically adapted for coprophagy. In this study, we treated all species from the Scarabaeinae as coprophagous, including the fungivorous *Coptorhina*, which was in the basal position of the Scarabaeinae. Plant-eating scarabs (e.g., Cetoniinae, Dynastinae, Melolonthinae, Orphninae, Rutelinae, Glaphyridae, Lucanidae, and Passalidae) were treated as phytophagous in this study.

There are also occasionally dung feeders within the Aphodiinae, Hybosoridae and Geotrupidae, but their diets are not as restrictive as that of the Scarabaeinae. The family Geotrupidae primarily consists of detritivores, provisioning their nests with (often moldy) leaf litter. Species of the genus *Lethrus* within the Geotrupidae (e.g., *Lethrus apterus*), which cut and drag grass into their underground nests using their mandibles, can be a significant pest on grasslands (e.g., *Artemisia dalai*) [18]. Most hybosorides are known to feed on decomposing plant and animal matter. Larvae of the genus *Cryptogenius* live in rotten logs and feed on decayed wood and fungal hyphae [8]. The Aphodiinae are primarily saprophagous, feeding on dung, rotten wood and decaying plant matter. Some are known to frequent mammal burrows, and others are inquilines. Most ochodaeines feed on fungi [19], and the trogides are necrophagous [3]. As a group, the Glaresidae (of an unknown feeding type but likely similar to the Trogidae), which may eat dung-type soft food and also hard food, are treated as omnivorous, which covers necrophagy, mycophagy, detritivores, saprophagy, coprophagy, and other feeding types.

Eleven tribes (100 % of global Scarabaeinae tribes), 32 genera (approximately 13.3 % of global Scarabaeinae genera), and 72 Scarabaeinae species (approximately 1.2 % of global Scarabaeinae species) were included in the inner group. One or two species from each genus or subgenus were selected for the analyses. The chosen outgroups consisted of 115 species from the major lineages of the Scarabaeoidea for the phylogenetic and morphometric analyses [20–22].

### Micro-CT scanning and three-dimensional reconstruction

The mandibles from three specimens representing three feeding types were scanned with an X-radia 400 at the Institute of Zoology, Chinese Academy of Sciences (beam strength: 60KV, absorption contrast).

Micro-computed tomography (micro-CT) images were used for the 3D reconstructions. Based on the obtained image stacks, the structures of the specimens were reconstructed using Amira 5.4. The data files were then transferred to Geomagic Studio 12 to use the smoothing function and the specific display and rendering options available in this software. Final figures were prepared with Photoshop CS5 (Adobe).

### Geometric morphometric methods

Geometric morphometric analyses of mandible variation were based on a single curve (Fig. 1b). The curve was re-sampled in 50 semi-landmarks. Cartesian coordinates of the landmarks and curves were digitized with tps-DIG 2.05 [23], and the landmark configurations were scaled, translated, and rotated against the consensus configuration using the Procrustes superimposition method [24]. The shape differences of the mandibles among the three feeding types (omnivory, phytophagy and coprophagy) were inferred based on Principal Component Analysis (PCA) and Canonical Variate Analysis (CVA) in MorphoJ 1.06a [25] (Fig. 4). The average shapes of the mandible, which were treated as the terminal taxa in the phylogenetic combined analysis – at the sub- and family levels for outgroups and at the genus level for the inner group – were computed in MorphoJ 1.06a [25].

The phylogenetic relationships of each genus within the Scarabaeinae (inner groups, 32 genera in total) and each high-ranking taxon from the Scarabaeoidea (outgroups, 18 taxa in total) were reconstructed by revising the trees [20, 26, 27]. The comparative analyses in this study were based on the phylogenetic tree with the same taxon used in the geometric morphometrics. Landmark data were entered into Mesquite 2.72 [28] as a continuous matrix and linked to the phylogenetic tree. As branch lengths [29] were missing in this tree, we followed the evaluation method proposed by Klingenberg and Marugán-Lobón [30]. All branches were assigned an equal length (i.e., assuming an evolutionary model with the same expected degree of morphological change on every branch). The ancestral forms of all nodes were reconstructed using the traces of all characters and the landmark drawings from the modules of the Rhetenor package in Mesquite [28]. The ancestral states of all nodes were calculated and exported. The computed data for the nodes was integrated with the original landmark data in Excel and NTSYS-pc [31]. In this case, the shape differences in mandibles among extant and extinct scarabs were inferred based on PCA and CVA in MorphoJ 1.06a. The feeding types of ancestors (nodes) were mapped onto the phylogenetic tree based on either the Procrustes or the Mahalanobis distances (using the reciprocal transformation), which directly showed the ancestor’s possible feeding type. Ethical Approval was not required for this study.

## Results

### Mandible shape differences among three feeding types in the Scarabaeoidea

**Fig. 2:**
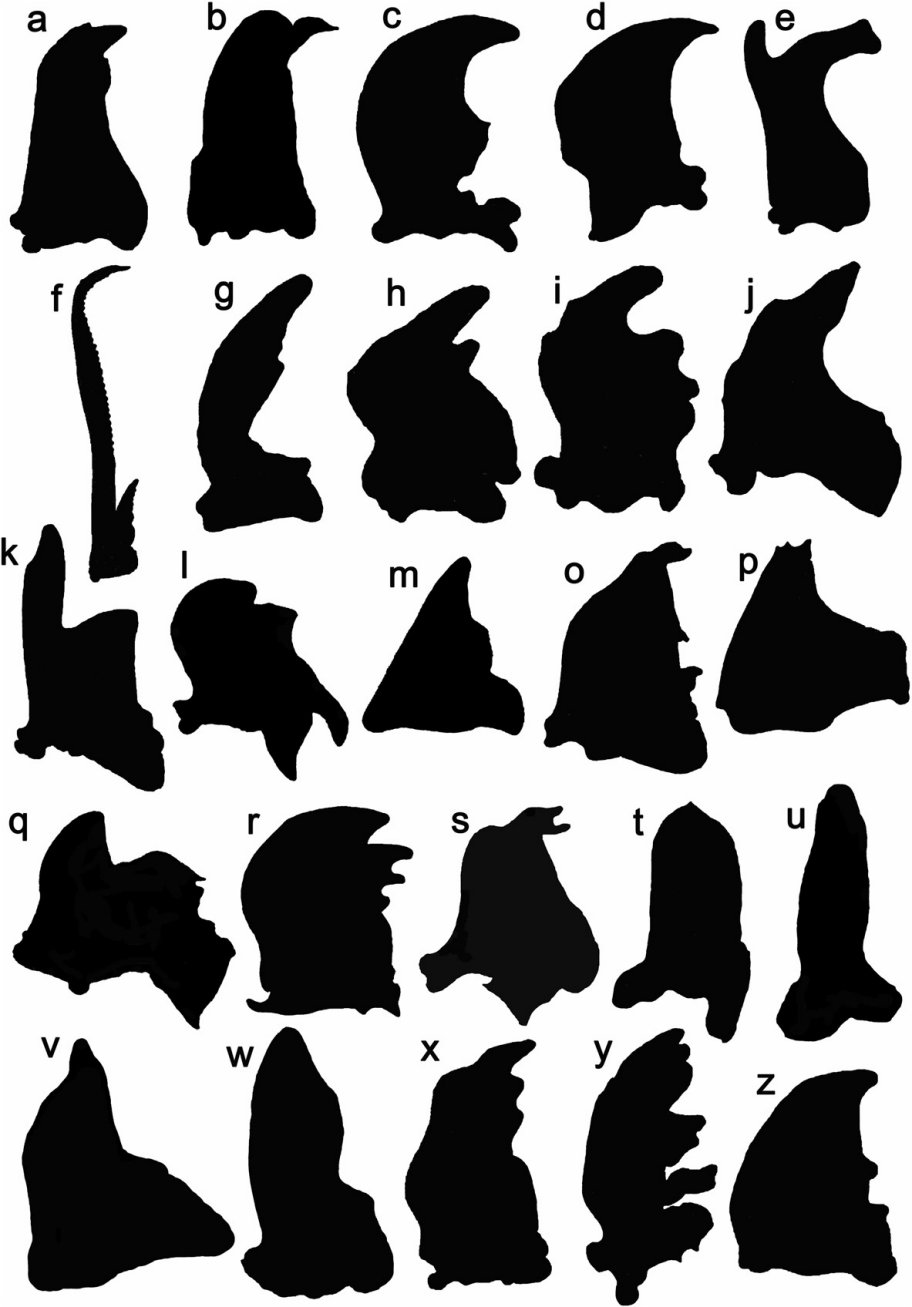
Mandibles of selected lineages from the Scarabaeoidea. **a.** Geotrupidae (*Anoplotrupes stercorosus*); **b.** Geotrupidae, Bolboceratinae (*Bolbocerastes serratus*); **c**. Hybosoridae (*Araeotanopus* sp.); **d.** Hybosoridae (*Metachaetodus discus*); **e.** Lucanidae (*Aesalus scarabaeoides*); **f.** Lucanidae (*Chinsognalhus grantii*); **g.** Ochodaeidae (*Enodognathus gilletti*); **h.** Ochodaeidae (*Pseudochodaeus estriatus*); **i.** Scarabaeidae, Aphodiinae, Aegialiini (*Eremazus unistriatus*); **j.** Scarabaeidae, Aphodiinae (*Drepanocanthus* sp.); **k.** Scarabaeidae, Cetoniinae (*Dicronorrhina* sp.); **l.** Scarabaeidae, Cetoniinae (*Clinterocera mandarina*); **m.** Scarabaeidae, Dynastinae (*Allomyrina dichotoma*); **o.** Scarabaeidae, Melolonthinae, Hopliini (*Hoplia* sp.); **p**. Scarabaeidae, Melolonthinae (*Clitopa* sp.); **q.** Scarabaeidae, Melolonthinae, Sericini (*Maladera orientalis*); **r.** Scarabaeidae, Orphnidae (*Orphnus* sp.); **s.** Scarabaeidae, Rutelinae (*Anomala* sp.); **t.** Scarabaeidae, Melolonthinae, Euchirini (*Cheirotonus jansoni*); **u**. Scarabaeidae, Scarabaeinae, Coprini (*Heliocopris dominus*); **v.** Scarabaeidae, Scarabaeinae, Eucraniini (*Eucranium arachnoides*); **w.** Scarabaeidae, Scarabaeinae, Coprini (*Synapsis yunnanus*); **x.** Glaresidae (*Glaresis impressicollis*); **y.** Passalidae (*Odontotaenius disjunctus*); **z.** Trogidae (*Trox monlanus*)

**Fig. 3:**
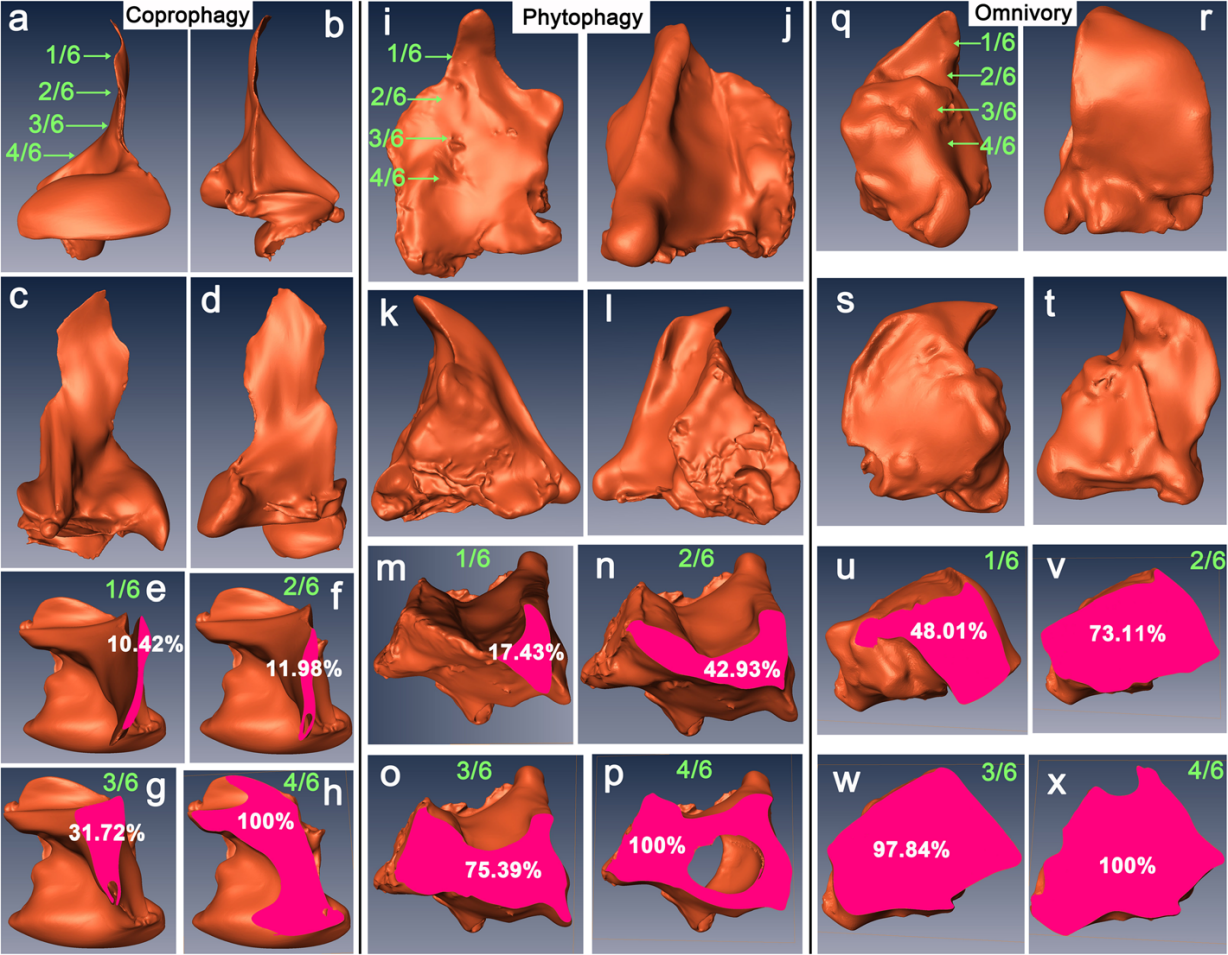
Morphological comparison of the mandible from three feeding types (omnivory, phytophagy and coprophagy) based on 3D models. Scarabaeinae: *Kheper devotus* (**a**–**h**); Dynastinae: *Allomyrina dichotoma* (**i**–**p**); Trogidae: *Trox* sp. (**q**–**x**). Lateral view (**a**, **b**, **i**, **j**, **q**, **r**); dorsal view (**c**, **k**, **s**); ventral view (**d**, **l**, **t**); cross-sections in different positions (**e**–**h**, **m**–**p**, **u**–**x**; percentages indicate the cross-sectional area of each position divided by the top 4/6 position)

Based on the comparative morphology approach, the mandible of the Scarabaeinae was characterized by a long blade-shape that was distally membranous and semi-transparent (Fig. 2). The mandibles of non-coprophagous scarabs were short (except the Lucanidae) and highly chitinized (Fig. 2). The distal portion of the coprophagous mandible was clearly transparent, while the non-coprophagous mandibles were not. Furthermore, the lateral view of the 3D models of the mandibles showed that the distal portion of the coprophagous mandible was significantly slimmer compared with the non-coprophagous mandibles (Fig. 3, Additional file 2). The virtual sectioning of the 3D models at four positions (the sample was divided into six equally sized sections and the top four proximal sections were analyzed) showed the mandibles of the three feeding types in cross-section (pink areas in Fig. 3 e–h, m–p, u–x). The slim distal portion of the coprophagous mandible was confirmed by the proportional cross-sectional areas of each position relative to the top 4/6 position, which were 10.42 % (top 1/6), 11.98 % (top 2/6) and 31.72 % (top 3/6). In comparison, the non-coprophagous mandibles were greatly broadened toward the base of the mandible; even the area of the top 1/6 cross-section could be 48.01 % of the top 4/6 cross-section in the Trogidae.

**Fig. 4:**
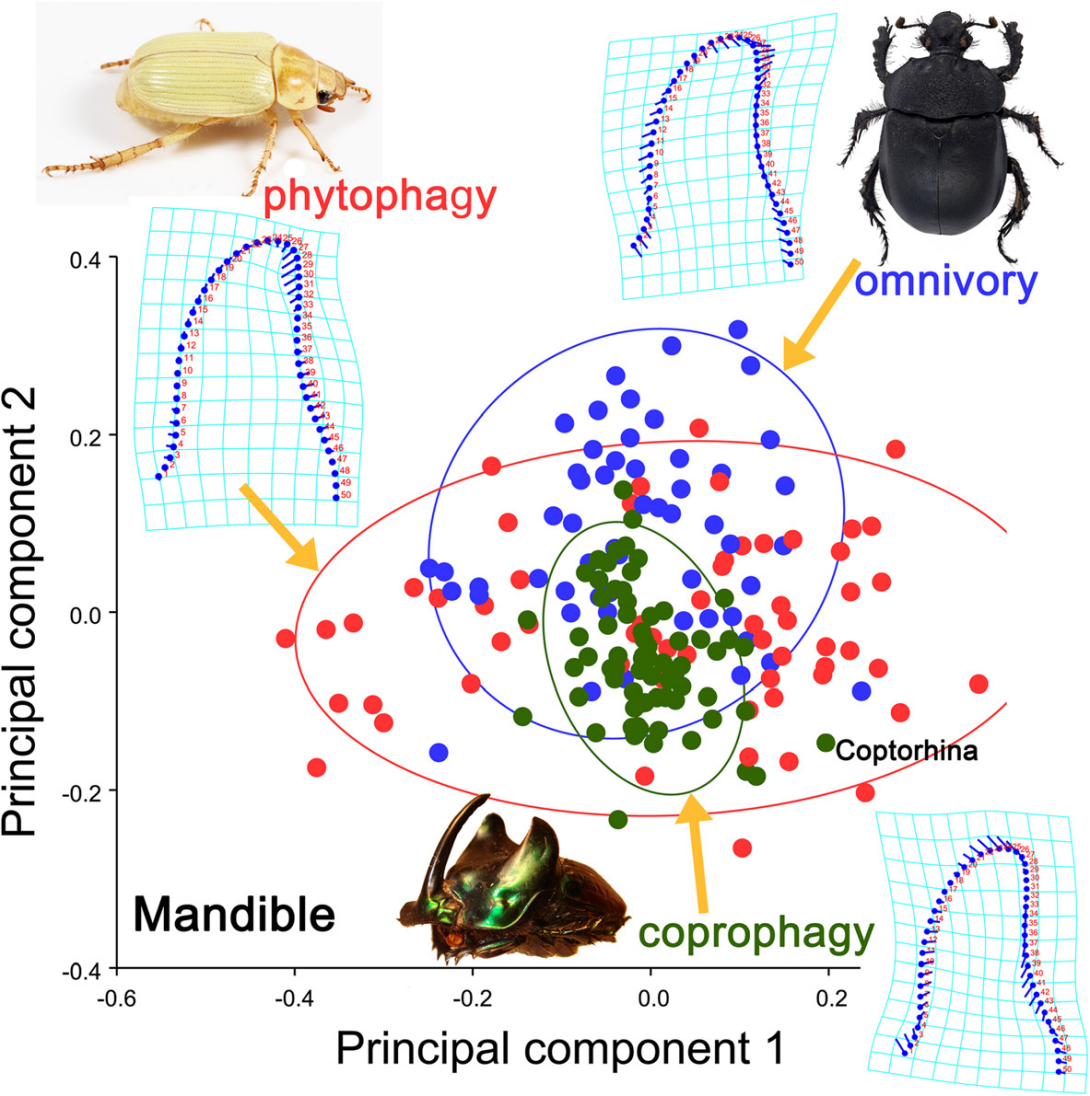
Shape differences of the mandible among the three feeding types (omnivory, phytophagy and coprophagy) based on Principal Component Analysis. The 90 % equal frequency ellipses containing approximately 90 % of the data points are shown. The *thin-plate splines* show the average shape of the mandible for each feeding type, corresponding to the deformation of the landmarks compared with the origin (the average mandibular shape of all species)

Based on the geometric morphometric approach, the first two relative warps of the mandible from 187 species accounted for 67.874 % of the variation among the species (Fig. 4). These warps were computed using a singular value decomposition of the weight matrix [32]. The first two relative warps were plotted to demonstrate variation along the two axes (Fig. 4). The average shape (Fig. 4, thin-plate splines) of the phytophagous mandible was characterized by the curved, sharp tip of the incisor. The incisor of the coprophagous mandible in the average shape (Fig. 4, thin-plate splines) was straight, and the tip pointed forward. Importantly, the coprophagous mandible was very long compared with the average mandibular shape of all species. The average shape (Fig. 4, thin-plate splines) of the omnivorous mandible showed that the tip was broad in the middle and pointed inward.

The greatest morphological variance in the mandible was observed in the phytophagous species (total variance = 0.06968641 computed from the PCA), whereas the least variance in the mandible was observed in coprophagous species (total variance = 0.01576603); the total variance for omnivorous species was 0.03690822. This result was consistent with the size of 90 % equal frequency ellipses, of which the ellipse of the coprophagous species was the smallest (Fig. 4).

Based on the above-mentioned differences from the PCA, a CVA was conducted that confirmed that mandible morphologies were significantly different among species belonging to the three feeding types. Mahalanobis and Procrustes distances among the groups were computed (Table 1). The *p*-values obtained with permutation tests (10,000 permutation rounds) for the Mahalanobis and Procrustes distances were all equal to or smaller than 0.0001, confirming that these distances were statistically significantly different.

**Table 1:** Canonical Variate Analysis (CVA) among the three feeding types

	OM vs. PH	OM vs. CO	PH vs. CO
Mahalanobis distances	4.7848	6.6525	5.2055
*p* value for Mahalanobis distances	<0.0001	<0.0001	<0.0001
Procrustes distances	0.1221	0.1610	0.10064
*p* value for Procrustes distances	0.0001	<0.0001	<0.0001

Feeding type: *OM* omnivory, *PH* phytophagy, *CO* coprophagy

### Mandible evolution in the Scarabaeinae and major lineages of the Scarabaeoidea

**Fig. 5:**
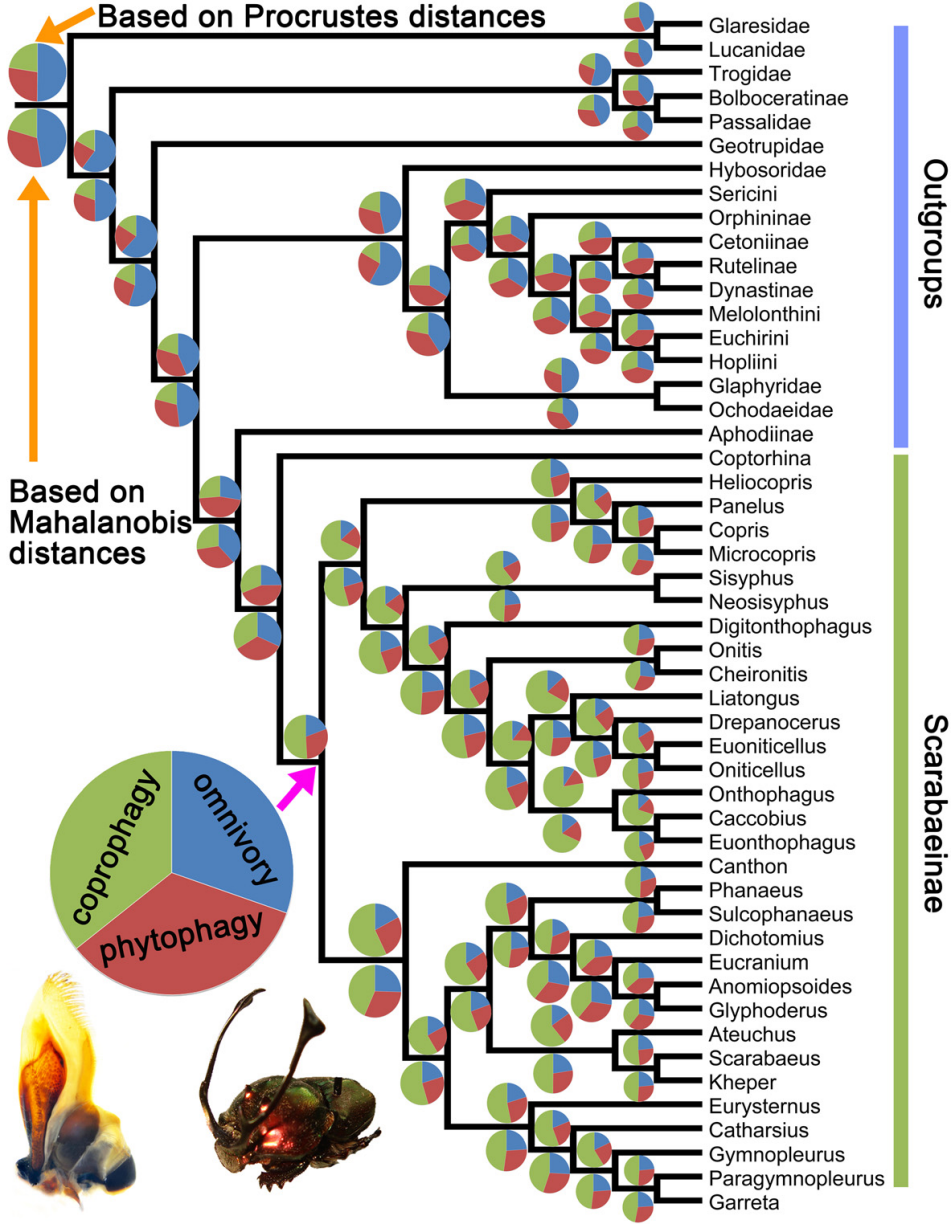
Possible feeding types of the ancestors of Scarabaeinae and the major lineages of Scarabaeoidea based on the reconstruction of mandible morphology

**Fig. 6:**
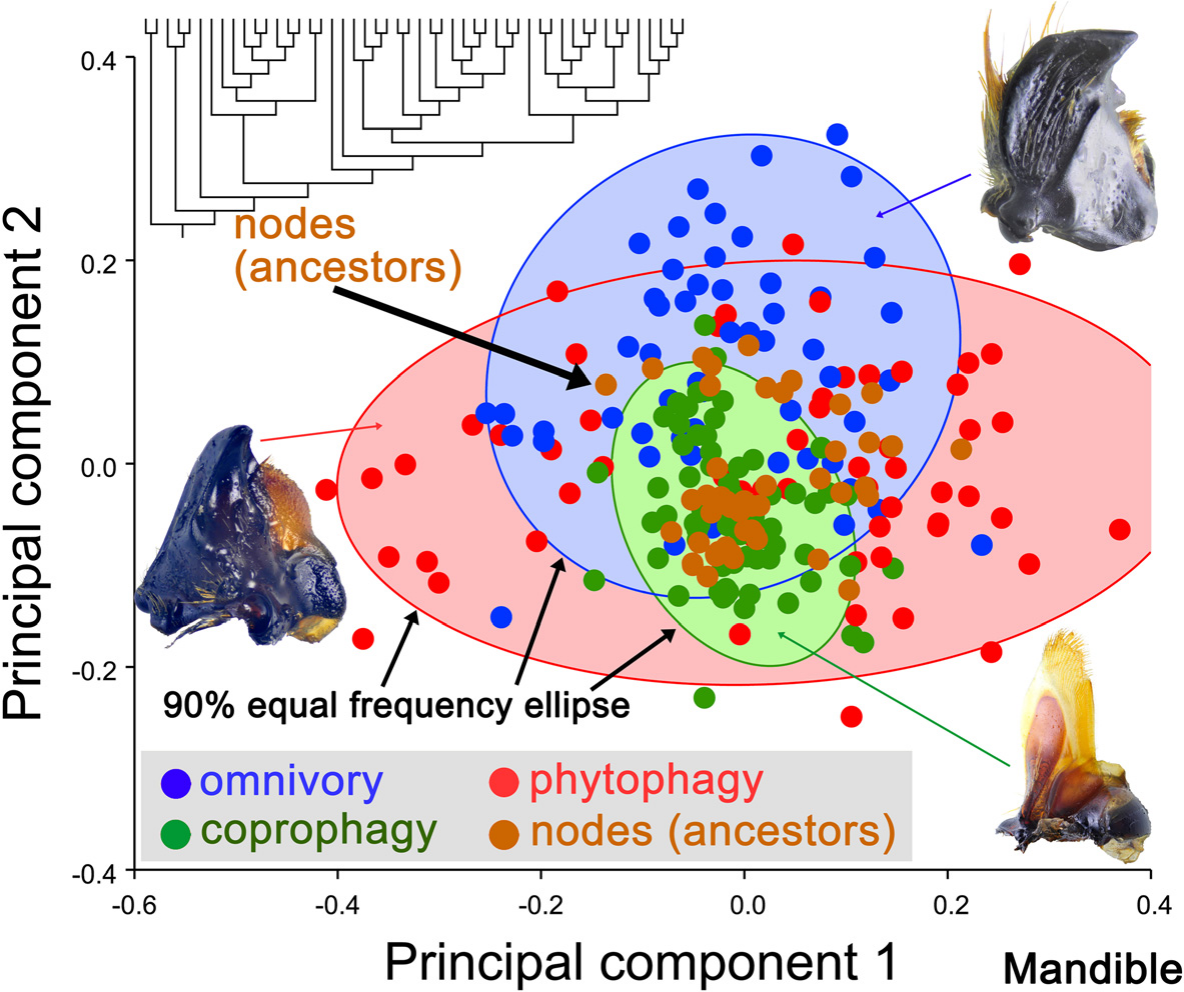
Mandibular shape differences among the extant and extinct taxa from the major Scarabaeoidea lineages based on Principal Component Analysis

The average shape of the mandible for each genus from the Scarabaeinae and each high-ranking taxon from the Scarabaeoidea was mapped onto a phylogenetic tree revised from previous studies [20, 26, 27] (Fig. 5, Additional file 1: Figure S1). The ancestral mandibular shapes (nodes) were computed and compared with modern species from the three feeding-type groups (Fig. 6). The qualitative result of the possible feeding type of the nodes is shown in a plot of the first two PCs (Fig. 6), in which the nodes were distributed in a 90 % equal frequency ellipse for each of the different feeding-type species groups. The quantitative result of the possible feeding type of the nodes was computed based on a CVA analysis of the shape differences between each ancestor (node) and the three feeding type species groups (Additional file 1: Table S2, Table S3). All ancestors of the scarabaeines, except the ancestor of the Scarabaeinae (node 38 in Additional file 1: Figure S1), were clearly coprophagous; this observation was supported by both the Procrustes and the Mahalanobis distances (Fig. 5, green [largest] section in the pie charts of related nodes; Additional file 1: Table S2, Table S3). Not all ancestors of phytophagous species were phytophagous; at least nodes 17 and 20 (Additional file 1: Figure S1) were not well supported as phytophagous (Fig. 5, red section in the pie charts of related nodes; Additional file 1: Table S2, Table S3), but instead appeared to be omnivorous. The nodes (nodes 2, 3, 6, 7, 12, 14, 36 in Additional file 1: Figure S1) near the root of the tree were largely supported as omnivorous.

## Discussion

### Morphological comparison of mandibles among the three feeding types within the Scarabaeoidea

The blade-shape of the coprophagous mandible is supported by the 3D reconstruction, in which the thickness of the distal portion of the coprophagous 3D model is not significantly changed. Based on our observations, the material property of the slim portion of the dung beetle’s mandible is very elastic compared with the non-coprophagous scarab. Proteiform mandibles are suitable for fitting tightly with other mouthparts, particularly the maxilla, to collect food particles from dung. However, the biting function is almost lost in these proteiform mandibles. This difference in functionality is also supported by a preliminary simulation using Finite Element Analysis (FEA), which demonstrates that the mandibles of the Scarabaeinae can be broken more easily than those of the Lucanidae when both Scarabaeinae and Lucanidae mandibular 3D models are set to the same material properties (i.e., those of the Lucanidae mandible) and the same boundary conditions (unpublished data). This adaptation could be associated with many benefits for dung feeding. With the aid of highly efficient and adaptative mouthparts, the Scarabaeinae dominate dung communities, which simultaneously support a wide variety of dung feeding-related behaviors.

The mandibular shape is very conservative in the Scarabaeinae, showing the least morphological variation among the three feeding groups (Fig. 4). The mandibles of most other dung-feeding scarabs are different from the Scarabaeinae. For example, the Aphodiinae, which are generally assumed to be the sister-group of the Scarabaeinae, is very different from the Scarabaeinae in terms of mandibular morphology [2, 20, 21, 27, 33, 34]. Most aphodiine feed on dung, humus, or even a “solid” diet. Although the food substrate for most of the Aphodiinae is dung (similar to the Scarabaeinae) the morphology of the mouthparts in these two groups does not strongly overlap. The aphodiine mandible is shorter and more robust than that of the Scarabaeinae (Fig. 2). These morphological differences could have been influenced by the different environments preferred by the species of these two groups. The environment not only affects the distribution of mammals and their food plants but also significantly influences the quantity, distribution, shape (mass or pellet-form), texture, nutritional quality, and the rate at which the condition of the dung changes, which could be key for the adaptation of mouthparts in both the Aphodiinae and the Scarabaeinae. For example, the nutrient, fiber, and water contents in the dung might depend on the species of vertebrate and the season and availability of food plants. Ruminants produce less fiber and finer dung than non-ruminants. In the rainy season, the water content of dung is higher than in the dry season. As dung beetles prefer fresh dung, normally within 48 h of defecation, the rate of change in the dung condition could be a factor influencing the tolerance of the accepted food substrate. Behaviorally, aphodiines are dwellers and are typically located in temperate regions, whereas scarabaeines are rollers or tunnelers, and most of the species live in tropical regions, where dung deposited on the ground changes rapidly to a hard and dry condition within a short time. In contrast, dung transported underground can be kept in a soft condition for a longer period of time. Although the Aphodiinae and Scarabaeinae co-exist in many regions, the niche divergence of both groups has clearly influenced the morphological evolution of mouthparts.

### Mandible evolution in the Scarabaeinae

The typical mandibles of coprophagous dung beetles, which could be used to process soft food and would consequently be less important for biting, may not have appeared in the very early evolutionary stages of the Scarabaeinae and Aphodiinae (Fig. 5), but instead may have occurred later, after the radiation of the Scarabaeinae. Our findings, based on geometric morphometric evidence, are consistent with the results from molecular phylogenetic analysis [20]. The ancestor of the Scarabaeinae and the Aphodiinae was in an evolutionary transition between processing more solid and softer substrates and may have been omnivorous (e.g., saprophagous), indicating that the mandibles of these ancestors were not typically coprophagous. This finding confirms the previous hypothesis [3, 4].

The mandibular morphology of the extinct Scarabaeinae was already highly adapted for soft food, as there were no significant differences between extinct and extant Scarabaeinae in this character system. Among the extinct species, the mandibles of the Scarabaeinae ancestor were marginally shorter and broader than in modern species, although they were still distinctly longer and narrower than in the modern Aphodiinae based on this study. The major differences in the mandibles between the two subgroups of Scarabaeinae, the *Onthophagus* lineage and the *Scarabaeus* lineage, are the width of the base and the shape of the molar lobe. The mandible of the ancestor of the *Onthophagus* lineage was narrower basally than in the ancestor of the *Scarabaeus* lineage. The molar lobe of the ancestor of the *Onthophagus* lineage is bent slightly downwards, whereas it is bent slightly upwards in the ancestor of the *Scarabaeus* lineages. The descendants of the two ancestral forms developed these characteristics to a more distinct degree. The functional adaptations of the morphological diversification of the width of the base and the shape of the molar lobe are not certain. Determining whether a wider mandibular base consumes more energy during the movement of mandible and the molar morphology could affect feeding efficiency requires additional work.

### Origin of coprophagy

The description of the oldest known Scarabaeinae fossil, *Cretonitis copripes*, was based on a fragment of legs from the Lower Cretaceous [35]. Morphologically, these legs could represent a species of the Scarabaeinae, but it cannot be excluded that they belong to a different group of Scarabaeoidea. Except for this uncertain fossil evidence and molecular dating, which places the crown group of the Scarabaeinae at approximately 86 to 100 Ma [20], a Late Cretaceous/Early Tertiary origin of the Scarabaeinae appears plausible [4, 26, 33, 36]. The oldest recorded fossil of the Aegialiini is from the Lower Cretaceous, whereas the oldest fossil of the Aphodiinae is known from the Upper Cretaceous [36]. Independent of the precise time of origin of the Scarabaeinae, Aphodiinae and Aegialiini, there is no doubt that the rise and rapid radiation of the Scarabaeinae was facilitated by the rapidly increasing and highly diversified dung resources provided by the explosive radiation of mammals in the Tertiary [1, 4, 20].

The Hybosoridae and Geotrupidae originated from the Jurassic [35–38], which is significantly older than the origin of the Scarabaeinae and mammals. In contrast to the specific adaptation to mammalian dung in the Scarabaeinae, the mouthparts of the Hybosoridae and Geotrupidae could feed on a wide variety of food textures, such as dung, carrion, humus, fungi, rotting wood, etc. Overall, coprophagy originated from omnivory, and very likely saprophagy, based on the mandibular evolution of dung-feeding scarabs. Furthermore, phytophagy may also have originated from omnivory.

## Additional files

Additional file 1:
**Table S1.** List of species examined. **Table S2.** Procrustes distances among the ancestors (nodes) and the groups from each of the three feeding types. **Table S3.** Mahalanobis distances among the ancestors (nodes) and the groups from each of the three feeding types. **Figure S1.** Phylogenetic relationships of the studied species with node numbers. (DOC 597 kb) Additional file 2: 3D mandible model in 3D PDF of the Scarabaeinae: *Kheper devotus*. 3D mandible model in 3D PDF of the Dynastinae: *Allomyrina dichotoma*. 3D mandible model in 3D PDF of the Trogidae: *Trox* sp. (ZIP 30884 kb)
