# Supplementary material for: Mandible evolution in the Scarabaeinae (Coleoptera: Scarabaeidae) and adaptations to coprophagous habits
Source: Front Zool. 2015 Oct 28;12:30. doi: 10.1186/s12983-015-0123-z (PMC4625450; doi:10.1186/s12983-015-0123-z)
Supplement: Additional file 1: — Table S1. List of species examined. Table S2. Procrustes distances among the ancestors (nodes) and the groups from each of the three feeding types. Table S3. Mahalanobis distances among the ancestors (nodes) and the groups from each of the three feeding types. Figure S1. Phylogenetic relationships of the studied species with node numbers. (DOC 597 kb) [file 12983_2015_123_MOESM1_ESM.doc]

Online Supplementary Material

**Mandible evolution in the Scarabaeinae (Coleoptera: Scarabaeidae) and adaptations to coprophagous habits**

**Additional Tables**

Table A1. List of species examined.

Table A2. Procrustes distances among the ancestors (nodes) and the groups from each of the three feeding types.

Table A3. Mahalanobis distances among the ancestors (nodes) and the groups from each of the three feeding types.

**Additional figure**

Fig. A1. Phylogenetic relationships of the studied species with node numbers.

**Additional files**

3D mandible model in 3D PDF of the Scarabaeinae: *Kheper devotus*.

3D mandible model in 3D PDF of the Dynastinae: *Allomyrina dichotoma*.

3D mandible model in 3D PDF of the Trogidae: *Trox* sp.

**Additional Tables**

**Table A1. List of species examined**

Feeding type: OM=omnivory; PH=phytophagy; CO=coprophagy

| **Family** | | **Sub. Fam.** | **Tribe** | **Species** | **Feed** |
| --- | --- | --- | --- | --- | --- |
| **1** | Scarabaeidae | Scarabaeinae | Ateuchini | *Ateuchus latus* | CO |
| **2** | Scarabaeidae | Scarabaeinae | Ateuchini | *Dichotomius pelamon* | CO |
| **3** | Scarabaeidae | Scarabaeinae | Ateuchini | *Coptorhina auspicata* | CO |
| **4** | Scarabaeidae | Scarabaeinae | Coprini | *Catharsius granulatus* | CO |
| **5** | Scarabaeidae | Scarabaeinae | Coprini | *Catharsius molossus* | CO |
| **6** | Scarabaeidae | Scarabaeinae | Coprini | *Copris hispanus* | CO |
| **7** | Scarabaeidae | Scarabaeinae | Coprini | *Copris lunaris* | CO |
| **8** | Scarabaeidae | Scarabaeinae | Coprini | *Heliocopris bucephalus* | CO |
| **9** | Scarabaeidae | Scarabaeinae | Coprini | *Heliocopris dominus* | CO |
| **10** | Scarabaeidae | Scarabaeinae | Coprini | *Microcopris apicepunctatus* | CO |
| **11** | Scarabaeidae | Scarabaeinae | Deltochilini | *Canthon histrio* | CO |
| **12** | Scarabaeidae | Scarabaeinae | Deltochilini | *Panelus assamensis* | CO |
| **13** | Scarabaeidae | Scarabaeinae | Eucraniini | *Anomiopsoides heteroclyta* | CO |
| **14** | Scarabaeidae | Scarabaeinae | Eucraniini | *Eucranium arachnoides* | CO |
| **15** | Scarabaeidae | Scarabaeinae | Eucraniini | *Eucranium planicolle* | CO |
| **16** | Scarabaeidae | Scarabaeinae | Eucraniini | *Glyphoderus centralis* | CO |
| **17** | Scarabaeidae | Scarabaeinae | Gymnopleurini | *Garreta morosus* | CO |
| **18** | Scarabaeidae | Scarabaeinae | Gymnopleurini | *Garreta mundus* | CO |
| **19** | Scarabaeidae | Scarabaeinae | Gymnopleurini | *Gymnopleurus aciculatus* | CO |
| **20** | Scarabaeidae | Scarabaeinae | Gymnopleurini | *Gymnopleurus flagellatus* | CO |
| **21** | Scarabaeidae | Scarabaeinae | Gymnopleurini | *Paragymnopleurus melanarius* | CO |
| **22** | Scarabaeidae | Scarabaeinae | Gymnopleurini | *Paragymnopleurus sinuatus* | CO |
| **23** | Scarabaeidae | Scarabaeinae | Oniticellini | *Euoniticellus fulvus* | CO |
| **24** | Scarabaeidae | Scarabaeinae | Oniticellini | *Euoniticellus pallipe* | CO |
| **25** | Scarabaeidae | Scarabaeinae | Oniticellini | *Eurysternus hypocrita* | CO |
| **26** | Scarabaeidae | Scarabaeinae | Oniticellini | *Ixodina* *runicus= Drepanocerus runicus* | CO |
| **27** | Scarabaeidae | Scarabaeinae | Oniticellini | *Tibiodrepanus* *sinicus= Drepanocerus sinicus* | CO |
| **28** | Scarabaeidae | Scarabaeinae | Oniticellini | *Liatongus gagatinus* | CO |
| **29** | Scarabaeidae | Scarabaeinae | Oniticellini | *Oniticellus cinctus* | CO |
| **30** | Scarabaeidae | Scarabaeinae | Oniticellini | *Oniticellus rahadmistus* | CO |
| **31** | Scarabaeidae | Scarabaeinae | Onitini | *Chironitis arrowi* | CO |
| **32** | Scarabaeidae | Scarabaeinae | Onitini | *Chironitis pamphlius* | CO |
| **33** | Scarabaeidae | Scarabaeinae | Onitini | *Onitis philemon* | CO |
| **34** | Scarabaeidae | Scarabaeinae | Onthophagini | *Caccobius (Caccobius) denticollis* | CO |
| **35** | Scarabaeidae | Scarabaeinae | Onthophagini | *Caccobius (Caccobius) jessoensis* | CO |
| **36** | Scarabaeidae | Scarabaeinae | Onthophagini | *Caccobius (Caccophilus) himalayanus* | CO |
| **37** | Scarabaeidae | Scarabaeinae | Onthophagini | *Digitonthophagus gazella* | CO |
| **38** | Scarabaeidae | Scarabaeinae | Onthophagini | *Euonthophagus amyntas* | CO |
| **39** | Scarabaeidae | Scarabaeinae | Onthophagini | *Euonthophagus gibbosus* | CO |
| **40** | Scarabaeidae | Scarabaeinae | Onthophagini | *Onthophagus (Altonthophagus) cupreiceps* | CO |
| **41** | Scarabaeidae | Scarabaeinae | Onthophagini | *Onthophagus (Altonthophagus) tibetanus* | CO |
| **42** | Scarabaeidae | Scarabaeinae | Onthophagini | *Onthophagus (Colobonthophagus) armatus* | CO |
| **43** | Scarabaeidae | Scarabaeinae | Onthophagini | *Onthophagus (Colobonthophagus) tragus* | CO |
| **44** | Scarabaeidae | Scarabaeinae | Onthophagini | *Onthophagus (Furconthophagus) dapcauensis* | CO |
| **45** | Scarabaeidae | Scarabaeinae | Onthophagini | *Onthophagus (Macronthophagus) diabolicus* | CO |
| **46** | Scarabaeidae | Scarabaeinae | Onthophagini | *Onthophagus (Macronthophagus) manipurensis* | CO |
| **47** | Scarabaeidae | Scarabaeinae | Onthophagini | *Onthophagus (Matashia) gracilipes* | CO |
| **48** | Scarabaeidae | Scarabaeinae | Onthophagini | *Onthophagus (Matashia) kuluensis* | CO |
| **49** | Scarabaeidae | Scarabaeinae | Onthophagini | *Onthophagus (Micronthophagus) hystrix* | CO |
| **50** | Scarabaeidae | Scarabaeinae | Onthophagini | *Onthophagus (Micronthophagus) vigilans* | CO |
| **51** | Scarabaeidae | Scarabaeinae | Onthophagini | *Onthophagus (Onthophagiellus) crassicollis* | CO |
| **52** | Scarabaeidae | Scarabaeinae | Onthophagini | *Onthophagus (Onthophagus) bivertex* | CO |
| **53** | Scarabaeidae | Scarabaeinae | Onthophagini | *Onthophagus (Onthophagus) taurus* | CO |
| **54** | Scarabaeidae | Scarabaeinae | Onthophagini | *Onthophagus (Palaeonthophagus) gibbulus* | CO |
| **55** | Scarabaeidae | Scarabaeinae | Onthophagini | *Onthophagus (Palaeonthophagus) vacca* | CO |
| **56** | Scarabaeidae | Scarabaeinae | Onthophagini | *O. (Paraphanaeomorphus) argyropygus* | CO |
| **57** | Scarabaeidae | Scarabaeinae | Onthophagini | *Onthophagus (Paraphanaeomorphus) trituber* | CO |
| **58** | Scarabaeidae | Scarabaeinae | Onthophagini | *Onthophagus (Parascatonomus) discedens* | CO |
| **59** | Scarabaeidae | Scarabaeinae | Onthophagini | *Onthophagus (Parascatonomus) funebris* | CO |
| **60** | Scarabaeidae | Scarabaeinae | Onthophagini | *Onthophagus (Phanaeomorphus) fodiens* | CO |
| **61** | Scarabaeidae | Scarabaeinae | Onthophagini | *Onthophagus (Phanaeomorphus) sycophanta* | CO |
| **62** | Scarabaeidae | Scarabaeinae | Onthophagini | *Onthophagus (Proagoderus) yunnanus* | CO |
| **63** | Scarabaeidae | Scarabaeinae | Phanaeini | *Phanaeus cambeforti* | CO |
| **64** | Scarabaeidae | Scarabaeinae | Phanaeini | *Phanaeus palaeno* | CO |
| **65** | Scarabaeidae | Scarabaeinae | Phanaeini | *Sulcophanaeus faunus* | CO |
| **66** | Scarabaeidae | Scarabaeinae | Scarabaeini | *Kheper devotus* | CO |
| **67** | Scarabaeidae | Scarabaeinae | Scarabaeini | *Kheper erichsoni* | CO |
| **68** | Scarabaeidae | Scarabaeinae | Scarabaeini | *Scarabaeus sacer* | CO |
| **69** | Scarabaeidae | Scarabaeinae | Scarabaeini | *Scarabaeus typhon* | CO |
| **70** | Scarabaeidae | Scarabaeinae | Sisyphini | *Neosisyphus bowringi* | CO |
| **71** | Scarabaeidae | Scarabaeinae | Sisyphini | *Neosisyphus spinipes* | CO |
| **72** | Scarabaeidae | Scarabaeinae | Sisyphini | *Sisyphus schaefferi* | CO |
| **73** | Scarabaeidae | Aphodiinae | Aegialiini | *Aegialia arenaria* | OM |
| **74** | Scarabaeidae | Aphodiinae | Aegialiini | *Eremazus unistriatus* | OM |
| **75** | Scarabaeidae | Aphodiinae | Aphodiini | *Aphodius denticulatus* | OM |
| **76** | Scarabaeidae | Aphodiinae | Aphodiini | *Aphodius denticulatus* | OM |
| **77** | Scarabaeidae | Aphodiidae | Proctophanini | *Drepanocanthus sp.* | OM |
| **78** | Scarabaeidae | Cetoniinae | Cetoniini | *Cetonia viridiopata* | PH |
| **79** | Scarabaeidae | Cetoniinae | Cremastochedini | *Clinterocera mandarina* | PH |
| **80** | Scarabaeidae | Cetoniinae | Cremastochedini | *Cymophorus pulchellus* | PH |
| **81** | Scarabaeidae | Cetoniinae | Goliathini | *Dicronorhina sp.* | PH |
| **82** | Scarabaeidae | Cetoniinae | Cetoniini | *Gametis jucunda* | PH |
| **83** | Scarabaeidae | Cetoniinae | Cremastochedini | *Genuches sp.* | PH |
| **84** | Scarabaeidae | Cetoniinae | Goliathini | *Neophadimus auzouxi* | PH |
| **85** | Scarabaeidae | Cetoniinae | Cremastochedini | *Campsiura javanica* | PH |
| **86** | Scarabaeidae | Cetoniinae | Cremastochedini | *Campsiura mirabilis* | PH |
| **87** | Scarabaeidae | Cetoniinae | Trichiini | *Campulipus sp.* | PH |
| **88** | Scarabaeidae | Cetoniinae | Trichiini | *Gnorimella sp.* | PH |
| **89** | Scarabaeidae | Cetoniinae | Trichiini | *Pantodinus sp.* | PH |
| **90** | Scarabaeidae | Cetoniinae | Trichiini | *Lasiotrichius succinctus* | PH |
| **91** | Scarabaeidae | Cetoniinae | Valgini | *Valgus californicus* | PH |
| **92** | Scarabaeidae | Dynastinae | Dynastini | *Allomyrina dichotoma* | PH |
| **93** | Scarabaeidae | Dynastinae | Dynastini | *Allomyrina dichotoma* | PH |
| **94** | Scarabaeidae | Dynastinae | Cyclocephalini | *Ancognatha sp.* | PH |
| **95** | Scarabaeidae | Dynastinae | Pentodontini | *Aphonides sp.* | PH |
| **96** | Scarabaeidae | Dynastinae | Pentodontini | *Temnorrhyncus sp.* | PH |
| **97** | Scarabaeidae | Melolonthinae | Hopliini | *Gymnolomma sp.* | PH |
| **98** | Scarabaeidae | Melolonthinae | Hopliini | *Hoplia sp.* | PH |
| **99** | Scarabaeidae | Melolonthinae | Hopliini | *Peritrichia sp.* | PH |
| **100** | Scarabaeidae | Melolonthinae | Tanyproctini | *Clitopa sp.* | PH |
| **101** | Scarabaeidae | Melolonthinae | Tanyproctini | *Eucyclophylla sp.* | PH |
| **102** | Scarabaeidae | Melolonthinae | Melolonthini | *Melolontha frater* | PH |
| **103** | Scarabaeidae | Melolonthinae | Tanyproctini | *Pseudachloa sp.* | PH |
| **104** | Scarabaeidae | Melolonthinae | Tanyproctini | *Scapanoclypeus sp.* | PH |
| **105** | Scarabaeidae | Melolonthinae | Tanyproctini | *Sparrmannia sp.* | PH |
| **106** | Scarabaeidae | Melolonthinae | Sericini | *Trochalus sp.* | PH |
| **107** | Scarabaeidae | Melolonthinae | Sericini | *Maladera orientalis* | PH |
| **108** | Scarabaeidae | Melolonthinae | Euchirini | *Cheirotonus jansoni* | PH |
| **109** | Scarabaeidae | Orphninae | Orphnini | *Orphnus sp.* | PH |
| **110** | Scarabaeidae | Rutelinae | Anomalini | *Anomala corpulenta* | PH |
| **111** | Scarabaeidae | Rutelinae | Anomalini | *Anomala sp.* | PH |
| **112** | Scarabaeidae | Rutelinae | Anoplognathini | *Anoplognathus sp.* | PH |
| **113** | Scarabaeidae | Rutelinae | Anomalini | *Popillia mutans* | PH |
| **114** | Geotrupidae | Geotrupinae | Geotrupini | *Anoplotrupes stercorosus* | OM |
| **115** | Geotrupidae | Bolboceratinae | Bolboceratini | *Bolbocerastes serratus* | OM |
| **116** | Geotrupidae | Bolboceratinae | Bolboceratini | *Bolbocerotex sp.* | OM |
| **117** | Geotrupidae | Geotrupinae | Taurocerastini | *Frickius varioiosus* | OM |
| **118** | Geotrupidae | Geotrupinae | Geotrupini | *Geotrupes sterlorarius* | OM |
| **119** | Geotrupidae | Geotrupinae | Lethrini | *Lethrus geminatus* | OM |
| **120** | Geotrupidae | Bolboceratinae | Bolboceratini | *Meridiobolbus sp.* | OM |
| **121** | Geotrupidae | Bolboceratinae | Bolboceratini | *Mimobolbus sp.* | OM |
| **122** | Geotrupidae | Geotrupinae | Athyreini | *Parathyreus trituberculatus* | OM |
| **123** | Geotrupidae | Bolboceratinae | Bolboceratini | *Prototrupes sp.* | OM |
| **124** | Geotrupidae | Geotrupinae | Geotrupini | *Geotrupes vernalis* | OM |
| **125** | Glaphyridae | - | - | *Amphicoma fairmairei* | PH |
| **126** | Glaphyridae | - | - | *Amphicoma vulpes* | PH |
| **127** | Glaphyridae | - | - | *Lichnanthe apina* | PH |
| **128** | Glaresidae | - | - | *Glaresis impressicollis* | OM |
| **129** | Glaresidae | - | - | *Glaresis sp.* | OM |
| **130** | Hybosoridae | Anaidinae | - | *Anaides fossulatus* | OM |
| **131** | Hybosoridae | Hybosorinae | - | *Apalonychus waterhousei* | OM |
| **132** | Hybosoridae | Hybosorinae | - | *Araeotanopus sp.* | OM |
| **133** | Hybosoridae | Pachyplectrinae | - | *Brenskea testaceus* | OM |
| **134** | Hybosoridae | Ceratocanthinae | Ceratocanthini | *Acanthocerodes singularis* | OM |
| **135** | Hybosoridae | Ceratocanthinae | Ceratocanthini | *Ceratocanthus nitidus* | OM |
| **136** | Hybosoridae | Ceratocanthinae | Ceratocanthini | *Cloeotus aphodioides* | OM |
| **137** | Hybosoridae | Ceratocanthinae | Ceratocanthini | *Philharmostes zuluensis* | OM |
| **138** | Hybosoridae | Ceratocanthinae | Ceratocanthini | *Pterorthochaetes haroldi* | OM |
| **139** | Hybosoridae | Anaidinae | - | *Chaetodus piceus* | OM |
| **140** | Hybosoridae | Hybosorinae | - | *Coilodes gibbus* | OM |
| **141** | Hybosoridae | Anaidinae | - | *Cryptogenius miersianus* | OM |
| **142** | Hybosoridae | Hybosorinae | - | *Dicraeodon fimbriatus* | OM |
| **143** | Hybosoridae | Hybosorinae | - | *Hybosorus illigeri* | OM |
| **144** | Hybosoridae | Liparochrinae | - | *Liparochrus matthewsi* | OM |
| **145** | Hybosoridae | Hybosorinae | - | *Metachaetodus discus* | OM |
| **146** | Hybosoridae | Pachyplectrinae | - | *Pachyplectris laevis* | OM |
| **147** | Hybosoridae | Hybosorinae | - | *Phaeochrous mashunus* | OM |
| **148** | Hybosoridae | Hybosorinae | - | *Phaeochrous sp* | OM |
| **149** | Hybosoridae | Hybosorinae | - | *Phaeochridius uniformis* | OM |
| **150** | Hybosoridae | Hybosorinae | - | *Phaeocroops gilletti* | OM |
| **151** | Lucanidae | Lucaninae | Aegini | *Aegus taurus* | PH |
| **152** | Lucanidae | Aesalinae | Aesalini | *Aesalus scarabaeoides* | PH |
| **153** | Lucanidae | Aesalinae | Ceratognathini | *Ceratognathus sp.* | PH |
| **154** | Lucanidae | Syndesinae | Ceruchini | *Ceruchus chrysomelinus* | PH |
| **155** | Lucanidae | Lucaninae | Chiasognathini | *Chiasognathus grantii* | PH |
| **156** | Lucanidae | Lucaninae | Dorcini | *Cyclommatus multidentatus* | PH |
| **157** | Lucanidae | Lucaninae | Dorcini | *Cyclommatus zuberi* | PH |
| **158** | Lucanidae | Lucaninae | Dorcini | *Dorcus paralleloplpedus* | PH |
| **159** | Lucanidae | Lucaninae | Dorcini | *Dorcus bucephalus* | PH |
| **160** | Lucanidae | Lucaninae | Lucanini | *Hexarthrius buqueti* | PH |
| **161** | Lucanidae | Lampriminae | - | *Lamprima sp.* | PH |
| **162** | Lucanidae | Lucaninae | Lucanini | *Lucanus angusticornis* | PH |
| **163** | Lucanidae | Lucaninae | Lucanini | *Lucanus capreolus* | PH |
| **164** | Lucanidae | Lucaninae | Cladognathini | *Metopodontus bison* | PH |
| **165** | Lucanidae | Lucaninae | Figulini | *Nigidius sp.* | PH |
| **166** | Lucanidae | Lucaninae | Odontolabini | *Odontolabis sinensis* | PH |
| **167** | Lucanidae | Lucaninae | Odontolabini | *Odontolabis bellicosus* | PH |
| **168** | Lucanidae | Lucaninae | Cladognathini | *Prosopocoilus sp.* | PH |
| **169** | Lucanidae | Syndesinae | Sinodendronini | *Sinodendron cylindricum* | PH |
| **170** | Ochodaeidae | Chaetocanthinae | Chaetocanthini | *Chaetocanthus bechuanus* | OM |
| **171** | Ochodaeidae | Ochodaeinae | Ochodaeini | *Codocera ferruginea* | OM |
| **172** | Ochodaeidae | Ochodaeinae | Enodognathini | *Enodognathus gilletti* | OM |
| **173** | Ochodaeidae | Chaetocanthinae | Chaetocanthini | *Namibiotalpa fossilis* | OM |
| **174** | Ochodaeidae | Ochodaeinae | Ochodaeini | *Ochodaeus congoensis* | OM |
| **175** | Ochodaeidae | Ochodaeinae | Ochodaeini | *Ochodaeus gnatho* | OM |
| **176** | Ochodaeidae | Ochodaeinae | Ochodaeini | *Ochodaeus maculatus* | OM |
| **177** | Ochodaeidae | Ochodaeinae | Ochodaeini | *Ochodaeus serrugineus* | OM |
| **178** | Ochodaeidae | Ochodaeinae | Ochodaeini | *Ochodaeus xanthomelas* | OM |
| **179** | Ochodaeidae | Ochodaeinae | Enodognathini | *Odontochodaeus maxillosus* | OM |
| **180** | Ochodaeidae | Chaetocanthinae | Pseudochodaeini | *Pseudochodaeus estriatus* | OM |
| **181** | Ochodaeidae | Chaetocanthinae | Synochodaeini | *Synochodaeus cucullis* | OM |
| **182** | Passalidae | Macrolininae | - | *Didimus sansibaricus* | PH |
| **183** | Passalidae | Passalinae | Proculini | *Odontotaenius disjunctus* | PH |
| **184** | Trogidae | Omorginae | - | *Omorgus melancholicus* | OM |
| **185** | Trogidae | Omorginae | - | *Polynoncus bullatus* | OM |
| **186** | Trogidae | Troginae | - | *Trox cadareinus* | OM |
| **187** | Trogidae | Troginae | - | *Trox monlanus* | OM |

**Table A2. Procrustes distances among the ancestors (nodes) and the groups from each three feeding type**

Node numbers see Figure s1.

| **Node**  **No.** | **Omnivory** | **Phytophagy** | **Coprophagy** | **After data transformation (Reciprocal)** | | |
| --- | --- | --- | --- | --- | --- | --- |
|  |  |  |  | **Omnivory** | **Phytophagy** | **Coprophagy** |
| **2** | 0.0815 | 0.1527 | 0.188 | 12.26994 | 6.548788474 | 5.319149 |
| **3** | 0.1256 | 0.1823 | 0.2022 | 7.961783 | 5.485463522 | 4.945598 |
| **6** | 0.0493 | 0.1302 | 0.1816 | 20.28398 | 7.680491551 | 5.506608 |
| **7** | 0.0684 | 0.1323 | 0.1959 | 14.61988 | 7.558578987 | 5.104645 |
| **9** | 0.14 | 0.154 | 0.219 | 7.142857 | 6.493506494 | 4.56621 |
| **12** | 0.0437 | 0.121 | 0.1707 | 22.8833 | 8.26446281 | 5.858231 |
| **14** | 0.0724 | 0.0872 | 0.1543 | 13.81215 | 11.46788991 | 6.480881 |
| **15** | 0.0721 | 0.1022 | 0.1618 | 13.86963 | 9.784735812 | 6.18047 |
| **17** | 0.128 | 0.1043 | 0.174 | 7.8125 | 9.587727709 | 5.747126 |
| **18** | 0.2617 | 0.2021 | 0.2629 | 3.821169 | 4.948045522 | 3.803728 |
| **20** | 0.1711 | 0.1502 | 0.2138 | 5.844535 | 6.657789614 | 4.677268 |
| **22** | 0.1704 | 0.1133 | 0.1695 | 5.868545 | 8.826125331 | 5.899705 |
| **23** | 0.1997 | 0.1072 | 0.1617 | 5.007511 | 9.328358209 | 6.184292 |
| **25** | 0.1902 | 0.1082 | 0.1516 | 5.257624 | 9.242144177 | 6.596306 |
| **28** | 0.1944 | 0.1392 | 0.1832 | 5.144033 | 7.183908046 | 5.458515 |
| **30** | 0.1779 | 0.1124 | 0.1218 | 5.621135 | 8.896797153 | 8.210181 |
| **33** | 0.0573 | 0.0916 | 0.1479 | 17.45201 | 10.91703057 | 6.761325 |
| **36** | 0.1467 | 0.0883 | 0.1543 | 6.816633 | 11.32502831 | 6.480881 |
| **38** | 0.1791 | 0.1015 | 0.1423 | 5.583473 | 9.852216749 | 7.027407 |
| **40** | 0.1502 | 0.0814 | 0.0514 | 6.65779 | 12.28501229 | 19.45525 |
| **41** | 0.1612 | 0.111 | 0.0314 | 6.203474 | 9.009009009 | 31.84713 |
| **42** | 0.1885 | 0.1448 | 0.0701 | 5.30504 | 6.906077348 | 14.26534 |
| **44** | 0.1956 | 0.1286 | 0.0475 | 5.112474 | 7.776049767 | 21.05263 |
| **46** | 0.182 | 0.1342 | 0.0729 | 5.494505 | 7.451564829 | 13.71742 |
| **49** | 0.1625 | 0.1196 | 0.0342 | 6.153846 | 8.361204013 | 29.23977 |
| **50** | 0.156 | 0.1249 | 0.0451 | 6.410256 | 8.006405124 | 22.17295 |
| **53** | 0.1759 | 0.13 | 0.0517 | 5.685048 | 7.692307692 | 19.34236 |
| **55** | 0.1707 | 0.1221 | 0.0514 | 5.858231 | 8.19000819 | 19.45525 |
| **56** | 0.1794 | 0.1356 | 0.0883 | 5.574136 | 7.374631268 | 11.32503 |
| **59** | 0.1669 | 0.1131 | 0.0234 | 5.991612 | 8.84173298 | 42.73504 |
| **60** | 0.1844 | 0.1198 | 0.0348 | 5.422993 | 8.347245409 | 28.73563 |
| **62** | 0.1928 | 0.1233 | 0.0462 | 5.186722 | 8.110300081 | 21.64502 |
| **64** | 0.1883 | 0.1201 | 0.0496 | 5.310674 | 8.326394671 | 20.16129 |
| **67** | 0.1512 | 0.1076 | 0.0187 | 6.613757 | 9.293680297 | 53.47594 |
| **69** | 0.1469 | 0.1089 | 0.0275 | 6.807352 | 9.182736455 | 36.36364 |
| **72** | 0.1942 | 0.1229 | 0.0581 | 5.149331 | 8.136696501 | 17.2117 |
| **74** | 0.1963 | 0.1319 | 0.0557 | 5.094244 | 7.581501137 | 17.95332 |
| **75** | 0.1855 | 0.117 | 0.0477 | 5.390836 | 8.547008547 | 20.96436 |
| **76** | 0.1913 | 0.1179 | 0.0639 | 5.227392 | 8.481764207 | 15.64945 |
| **77** | 0.2016 | 0.1334 | 0.0817 | 4.960317 | 7.496251874 | 12.2399 |
| **80** | 0.195 | 0.1116 | 0.0764 | 5.128205 | 8.960573477 | 13.08901 |
| **82** | 0.2239 | 0.1289 | 0.1401 | 4.46628 | 7.757951901 | 7.137759 |
| **84** | 0.2626 | 0.1631 | 0.1737 | 3.808073 | 6.131207848 | 5.757052 |
| **87** | 0.1728 | 0.108 | 0.0416 | 5.787037 | 9.259259259 | 24.03846 |
| **89** | 0.1257 | 0.1142 | 0.058 | 7.955449 | 8.756567426 | 17.24138 |
| **92** | 0.2152 | 0.1611 | 0.0811 | 4.64684 | 6.207324643 | 12.33046 |
| **94** | 0.2302 | 0.1701 | 0.0784 | 4.344049 | 5.485463522 | 12.7551 |
| **96** | 0.2044 | 0.1551 | 0.0635 | 4.892368 | 6.447453256 | 15.74803 |
| **98** | 0.188 | 0.1605 | 0.092 | 5.319149 | 6.230529595 | 10.86957 |

**Table A3. Mahalanobis** **distances among the ancestors (nodes) and the groups from each three feeding type**

Node numbers see Figure s1.

| **Node**  **No.** | **Omnivory** | **Phytophagy** | **Coprophagy** | **After data transformation (Reciprocal)** | | |
| --- | --- | --- | --- | --- | --- | --- |
|  |  |  |  | **Omnivory** | **Phytophagy** | **Coprophagy** |
| **2** | 3.0939 | 4.5018 | 7.1278 | 0.323217 | 0.222133369 | 0.140296 |
| **3** | 3.9236 | 4.8096 | 7.2678 | 0.254868 | 0.207917498 | 0.137593 |
| **6** | 2.8509 | 4.6531 | 7.2427 | 0.350766 | 0.21491049 | 0.13807 |
| **7** | 4.4903 | 5.6744 | 7.9836 | 0.222702 | 0.176230086 | 0.125257 |
| **9** | 7.1924 | 7.225 | 8.9633 | 0.139036 | 0.138408304 | 0.111566 |
| **12** | 2.4626 | 4.853 | 7.2067 | 0.406075 | 0.206058108 | 0.13876 |
| **14** | 2.8883 | 4.4785 | 6.6197 | 0.346224 | 0.223289048 | 0.151064 |
| **15** | 1.8518 | 4.1395 | 6.436 | 0.540015 | 0.241575069 | 0.155376 |
| **17** | 3.6332 | 3.9908 | 6.747 | 0.275239 | 0.250576326 | 0.148214 |
| **18** | 6.8784 | 6.3448 | 8.8153 | 0.145383 | 0.157609381 | 0.113439 |
| **20** | 10.1831 | 9.8756 | 11.4561 | 0.098202 | 0.10125967 | 0.08729 |
| **22** | 7.974 | 7.2686 | 9.0263 | 0.125408 | 0.137578076 | 0.110787 |
| **23** | 5.8836 | 3.896 | 6.417 | 0.169964 | 0.256673511 | 0.155836 |
| **25** | 5.4823 | 3.4981 | 5.9501 | 0.182405 | 0.285869472 | 0.168064 |
| **28** | 5.6123 | 3.6597 | 6.4055 | 0.17818 | 0.273246441 | 0.156116 |
| **30** | 6.4376 | 4.5078 | 6.2556 | 0.155337 | 0.221837704 | 0.159857 |
| **33** | 3.6118 | 3.7138 | 6.3584 | 0.27687 | 0.269265981 | 0.157272 |
| **36** | 4.5483 | 5.0285 | 6.49 | 0.219862 | 0.198866461 | 0.154083 |
| **38** | 6.8201 | 6.3657 | 6.4257 | 0.146625 | 0.157091914 | 0.155625 |
| **40** | 6.9968 | 6.1443 | 5.0799 | 0.142922 | 0.16275247 | 0.196854 |
| **41** | 6.8882 | 5.6351 | 2.6039 | 0.145176 | 0.17745914 | 0.384039 |
| **42** | 7.8752 | 6.6645 | 3.3762 | 0.126981 | 0.150048766 | 0.296191 |
| **44** | 8.7985 | 7.4573 | 4.6627 | 0.113656 | 0.134096791 | 0.214468 |
| **46** | 9.7196 | 8.427 | 6.1768 | 0.102885 | 0.118666192 | 0.161896 |
| **49** | 7.4867 | 5.9693 | 2.5598 | 0.13357 | 0.16752383 | 0.390656 |
| **50** | 8.5445 | 7.0886 | 3.9906 | 0.117034 | 0.14107158 | 0.250589 |
| **53** | 7.8967 | 6.3417 | 3.6222 | 0.126635 | 0.157686425 | 0.276075 |
| **55** | 7.3302 | 6.007 | 2.9642 | 0.136422 | 0.166472449 | 0.337359 |
| **56** | 7.9156 | 7.0303 | 4.9499 | 0.126333 | 0.142241441 | 0.202024 |
| **59** | 7.1922 | 5.9215 | 2.4356 | 0.13904 | 0.168876129 | 0.410576 |
| **60** | 8.1747 | 7.0704 | 4.1297 | 0.122329 | 0.141434714 | 0.242148 |
| **62** | 7.8284 | 6.2809 | 3.0285 | 0.12774 | 0.159212852 | 0.330196 |
| **64** | 7.5532 | 6.186 | 3.1528 | 0.132394 | 0.161655351 | 0.317178 |
| **67** | 6.5855 | 5.349 | 1.3843 | 0.151849 | 0.186950832 | 0.722387 |
| **69** | 6.7363 | 5.6681 | 2.3163 | 0.148449 | 0.176425963 | 0.431723 |
| **72** | 7.5576 | 6.233 | 4.1945 | 0.132317 | 0.160436387 | 0.238407 |
| **74** | 7.3501 | 5.7947 | 2.6265 | 0.136053 | 0.172571488 | 0.380735 |
| **75** | 7.4838 | 5.7593 | 2.5092 | 0.133622 | 0.173632212 | 0.398533 |
| **76** | 7.8065 | 5.987 | 3.6458 | 0.128098 | 0.167028562 | 0.274288 |
| **77** | 7.5831 | 5.6261 | 3.6171 | 0.131872 | 0.177743019 | 0.276465 |
| **80** | 10.1548 | 8.622 | 7.199 | 0.098476 | 0.115982371 | 0.138908 |
| **82** | 9.4323 | 7.8001 | 6.5344 | 0.106019 | 0.128203485 | 0.153036 |
| **84** | 8.0575 | 6.128 | 4.8241 | 0.124108 | 0.163185379 | 0.207293 |
| **87** | 8.1497 | 6.6087 | 3.6386 | 0.122704 | 0.15131569 | 0.274831 |
| **89** | 7.9592 | 6.8457 | 3.794 | 0.125641 | 0.146077099 | 0.263574 |
| **92** | 8.2457 | 6.9628 | 4.1316 | 0.121275 | 0.143620383 | 0.242037 |
| **94** | 8.6545 | 7.6052 | 5.0036 | 0.115547 | 0.131488981 | 0.199856 |
| **96** | 8.3425 | 7.083 | 4.1495 | 0.119868 | 0.141183114 | 0.240993 |
| **98** | 8.5276 | 7.0281 | 4.3894 | 0.117266 | 0.142285966 | 0.227822 |

Additional figure


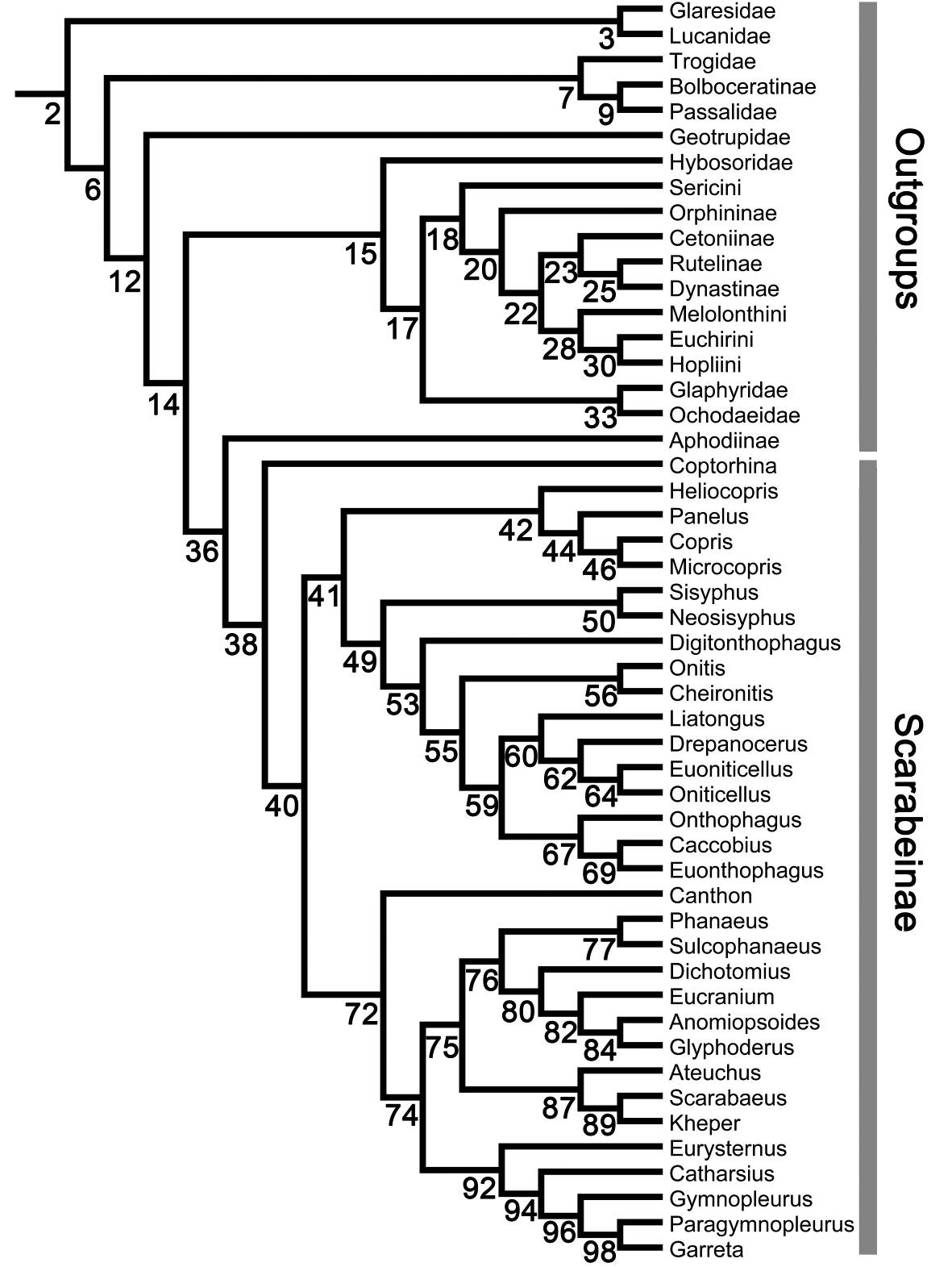


**Fig. A1. Phylogenetic relationships of the studied species with the node numbers.**
