# Supplementary figures and images for: Mandible evolution in the Scarabaeinae (Coleoptera: Scarabaeidae) and adaptations to coprophagous habits
Source: Front Zool. 2015 Oct 28;12:30. doi: 10.1186/s12983-015-0123-z (PMC4625450; doi:10.1186/s12983-015-0123-z)

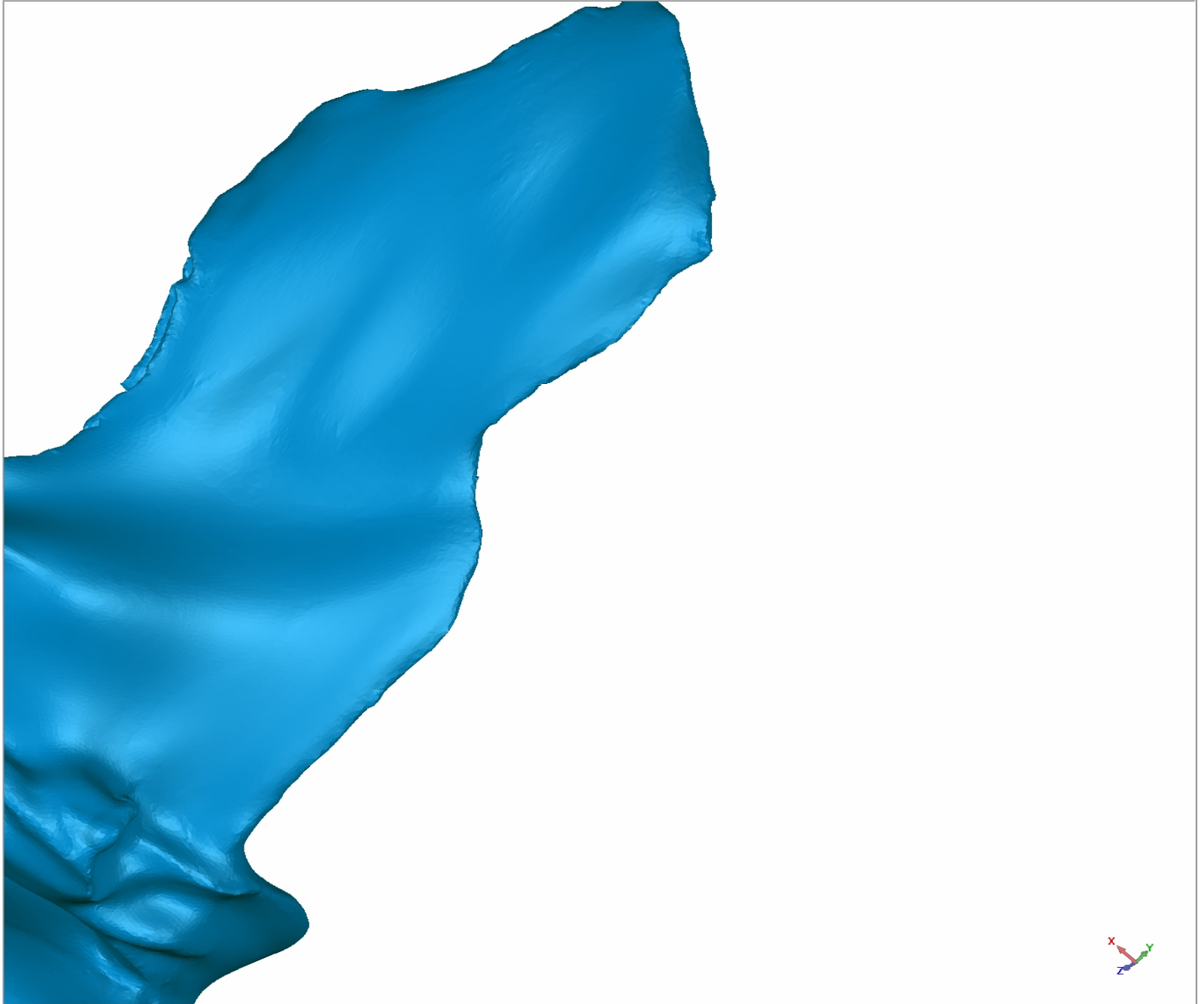

Click on the image to activate the 3D Model.

Supplement: Additional file 2: — 3D mandible model in 3D PDF of the Scarabaeinae: Kheper devotus. 3D mandible model in 3D PDF of the Dynastinae: Allomyrina dichotoma. 3D mandible model in 3D PDF of the Trogidae: Trox sp. (ZIP 30884 kb) [file 12983_2015_123_MOESM2_ESM.zip › Additional file 2/coprophagy-Scarabaeinae.pdf]

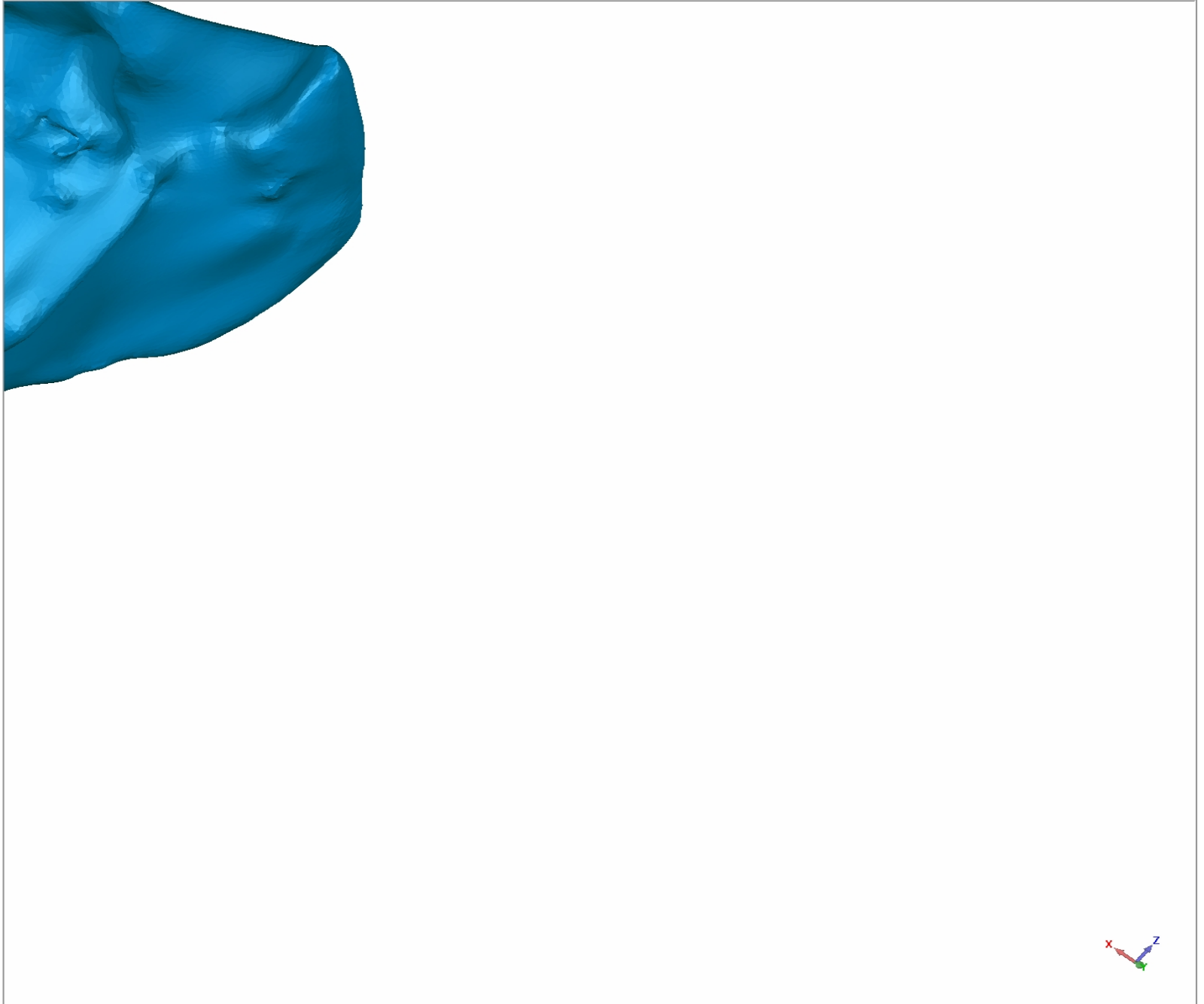

Click on the image to activate the 3D Model.

Supplement: Additional file 2: — 3D mandible model in 3D PDF of the Scarabaeinae: Kheper devotus. 3D mandible model in 3D PDF of the Dynastinae: Allomyrina dichotoma. 3D mandible model in 3D PDF of the Trogidae: Trox sp. (ZIP 30884 kb) [file 12983_2015_123_MOESM2_ESM.zip › Additional file 2/omnivory-Trogidae.pdf]

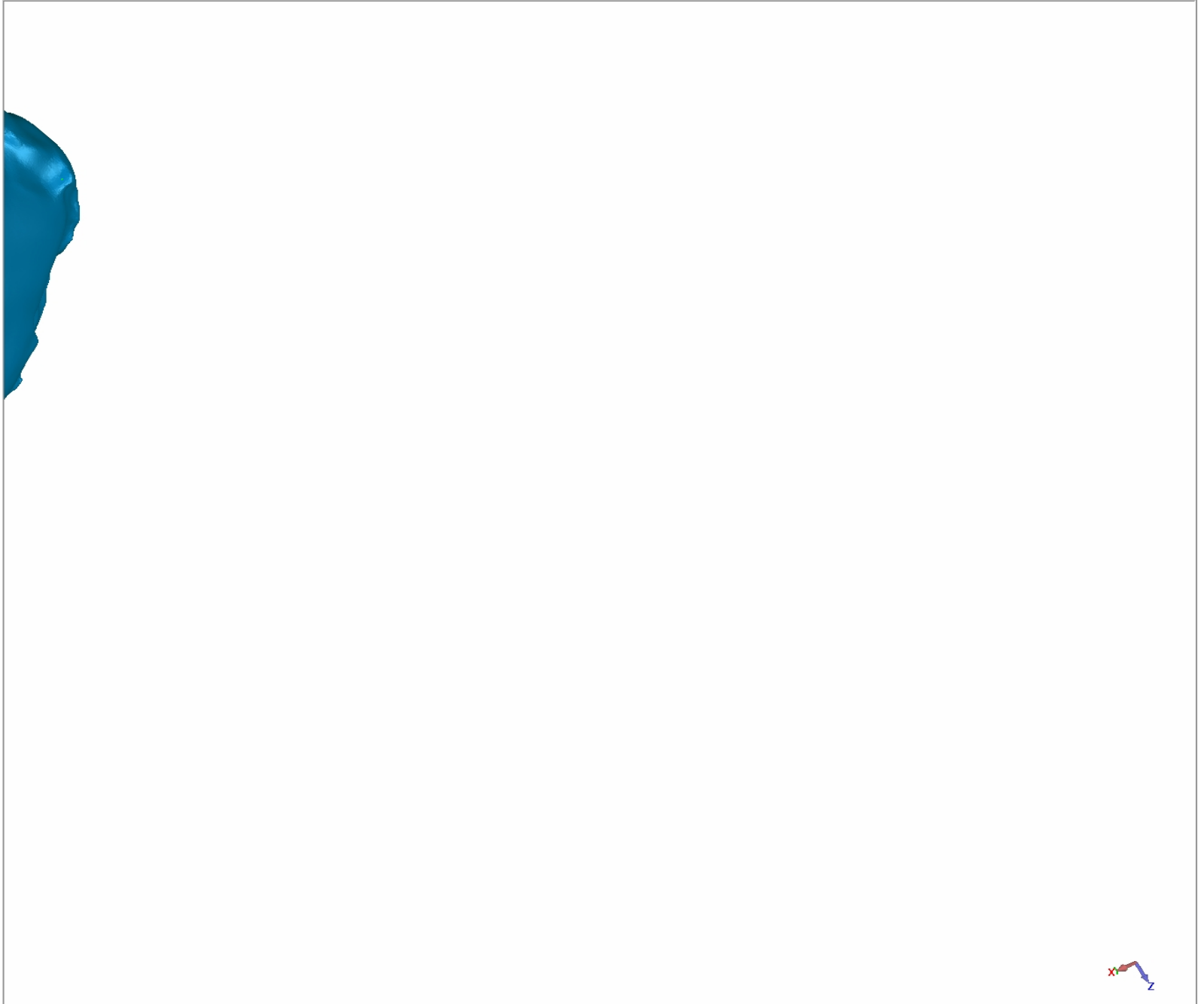

Click on the image to activate the 3D Model.

Supplement: Additional file 2: — 3D mandible model in 3D PDF of the Scarabaeinae: Kheper devotus. 3D mandible model in 3D PDF of the Dynastinae: Allomyrina dichotoma. 3D mandible model in 3D PDF of the Trogidae: Trox sp. (ZIP 30884 kb) [file 12983_2015_123_MOESM2_ESM.zip › Additional file 2/phytophagy-Dynastinae.pdf]
